# Treatment of Reactive Histiocytosis With Oclacitinib: A Retrospective Case Series of 10 Dogs

**DOI:** 10.1111/vde.70048

**Published:** 2026-01-28

**Authors:** Christine L. Cain, Andrew Lowe, Elizabeth A. Mauldin

**Affiliations:** ^1^ Department of Clinical Sciences and Advanced Medicine, School of Veterinary Medicine University of Pennsylvania Philadelphia Pennsylvania USA; ^2^ Capital City Specialty & Emergency Animal Hospital Kanata Ontario Canada; ^3^ Department of Pathobiology, School of Veterinary Medicine University of Pennsylvania Philadelphia Pennsylvania USA

## Abstract

**Background:**

Canine reactive histiocytosis is a proliferative disorder of activated interstitial dendritic cells with cutaneous and systemic forms. An immune‐mediated aetiology is likely, and systemic immunomodulatory agents such as corticosteroids, tetracycline/niacinamide, ciclosporin, azathioprine and leflunomide have been employed for its management.

**Hypothesis/Objectives:**

The aim of this retrospective case series is to report the clinical features and therapeutic response to oclacitinib in dogs with reactive histiocytosis.

**Animals:**

Ten privately owned dogs.

**Materials and Methods:**

All dogs were diagnosed with reactive histiocytosis based on compatible clinical history, skin lesions and histopathological features, and were treated with oclacitinib as a sole therapy. Immunohistochemical investigation was used to characterise dermal cellular infiltrates for eight of 10 dogs. Clinical features and case outcomes are summarised.

**Results:**

All 10 dogs presented with dermal nodules and/or erythematous plaques affecting the head, trunk or limbs; four dogs also had documented or suspected involvement of the nasal or oral cavities. Seven dogs had been treated previously with one or more immunomodulatory agents without durable disease control. All dogs were completely responsive to treatment with oclacitinib, at or slightly above the standard antipruritic dosage. Skin and mucosal lesions resolved within 2–12 weeks. Lesion remission was maintained with oclacitinib monotherapy for varying follow‐up times, although four dogs had brief recurrences addressed with adjustments in oclacitinib dosing.

**Conclusions and Clinical Relevance:**

Although reactive histiocytosis can have a naturally waxing and waning course, oclacitinib appears to be rapidly effective for management, even in cases refractory to other immunomodulatory agents or with involvement of the oral or nasal cavities.

**Animal Use Statement:**

Animal use was conducted in accordance with the international, national and institutional guidelines for the humane treatment of animals, and with relevant legislation.

## Graphical abstract

Background: Canine reactive histiocytosis is a proliferative disorder of activated interstitial dendritic cells with cutaneous and systemic forms. An immune‐mediated aetiology is likely, and systemic immunomodulatory agents such as corticosteroids, tetracycline/niacinamide, ciclosporin, azathioprine and leflunomide have been employed for its management. Hypothesis/Objectives: The aim of this retrospective case series is to report the clinical features and therapeutic response to oclacitinib in dogs with reactive histiocytosis. Conclusions and Clinical Relevance: Although reactive histiocytosis can have a naturally waxing and waning course, oclacitinib appears to be rapidly effective for management, even in cases refractory to other immunomodulatory agents or with involvement of the oral or nasal cavities.

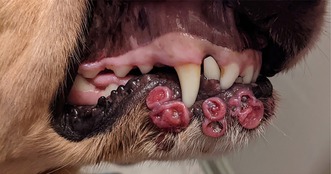

## 1 Introduction

Canine reactive histiocytosis is a non‐neoplastic proliferative disorder of activated interstitial dendritic cells. Affected dogs may have disease confined to the skin, subcutis and draining lymph nodes (cutaneous reactive histiocytosis), yet mucosal, ocular or visceral involvement (systemic reactive histiocytosis) can occur [1, 2]. Skin lesions (dermal to subcutaneous plaques and nodules with variable alopecia) present identically in both forms of canine reactive histiocytosis [1, 2, 3]. Definitive diagnosis of reactive histiocytosis requires compatible histological features—‘bottom‐heavy’ nodular‐to‐diffuse infiltrate of large histiocytes with variable numbers of small lymphocytes and neutrophils, which are most prominent within the mid‐ to deep dermis and subcutis—as well as exclusion of other differential diagnoses such as infectious agents and/or inflamed non‐epitheliotropic cutaneous T‐cell lymphoma [2, 3, 4]. Immunohistochemical stains can be performed to characterise histiocytes as interstitial dendritic cells, which express CD1a, CD11c/CD18, MHC class II, CD4, CD90 (Thy‐1) and Iba‐1, and not E‐cadherin [1, 2].

Immune dysregulation, particularly involving dendritic cell/T‐cell interactions and upregulation of pro‐inflammatory cytokines, is suspected to play a role in disease development, although the pathogenesis of reactive histiocytosis is not fully understood and a genetic predisposition also may contribute to disease development [1, 2, 3]. An antigenic trigger has been proposed yet has not been identified [1, 2]. An immune‐mediated aetiology is supported by the reported therapeutic response to immunomodulatory agents, including corticosteroids, tetracycline antibiotics in combination with niacinamide, azathioprine, ciclosporin and leflunomide [1, 2, 3].

Oclacitinib targets signalling of pro‐pruritic and pro‐inflammatory cytokines, including interleukin (IL)‐2, IL‐4, IL‐6, IL‐13 and IL‐31, via selective inhibition of Janus kinase (JAK) I [5]. Oclacitinib is labelled for control of pruritus in dogs secondary to allergic dermatitis, yet clinical efficacy as an immunomodulatory agent for management of a number of other immune‐mediated and autoimmune dermatoses has been reported [6, 7, 8, 9, 10, 11, 12, 13, 14]. The objective of this retrospective study is to report the clinical features and therapeutic response to oclacitinib of 10 dogs with reactive histiocytosis (characterised as cutaneous, unclassified or systemic, based on known or suspected involvement of the oral and/or nasal cavities).

## 2 Case Reports

Ten dogs with a diagnosis of reactive histiocytosis based on compatible clinical and histopathological features, and treated with oclacitinib, were identified. Medical records were searched for information regarding patient signalment, clinical features, treatment (prior therapies and oclacitinib) and treatment outcome. Dog owners were informed that oclacitinib was prescribed in an extra‐label manner for management of reactive histiocytosis before instituting treatment.

### 2.1 Signalment

Two mixed‐breed dogs and eight pure‐bred dogs were included in this case series. A single dog was represented from the following breeds: Bernese mountain dog, Doberman pinscher, Pembroke Welsh corgi, Shetland sheepdog, Labrador retriever and German shorthaired pointer. The remaining two dogs were golden retrievers. Five of the dogs were spayed females, and five were castrated males. The median age was 4 years (range 2–8 years), and the mean body weight was 31.5 kg (range 12.3–52.9 kg). The median duration of clinical signs at the initiation of oclacitinib therapy was 9.5 months (range 3 months–3 years). Table S1 summarises patient signalment and the duration of clinical signs at initiation of oclacitinib therapy for each individual dog.

### 2.2 Clinical Presentation

**FIGURE 1:**
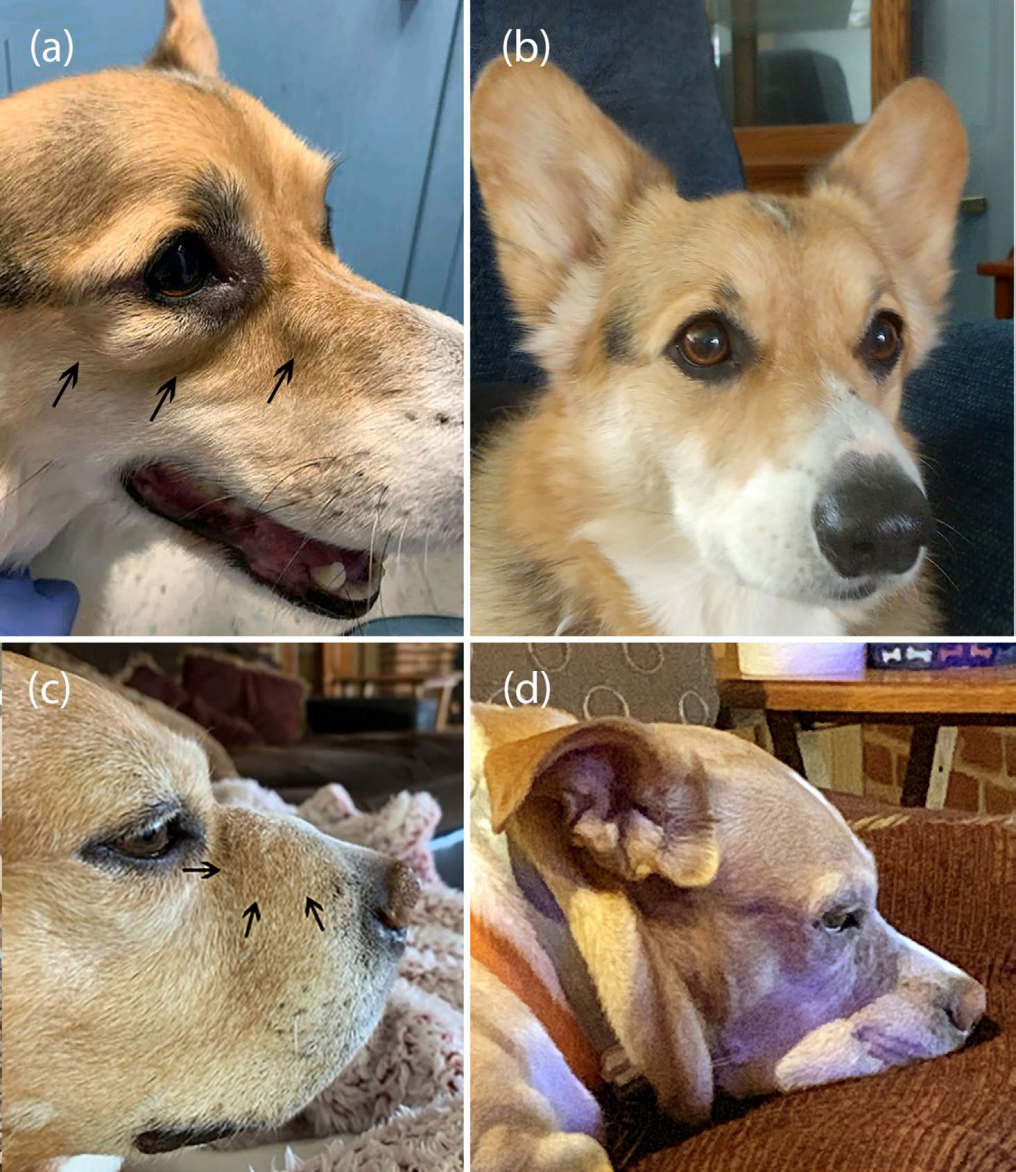
(a) Dog 4: Firm dermal nodules ventral to the right eye and on the right dorsolateral muzzle (arrows) before oclacitinib therapy; (b) Dog 4: Complete lesion resolution after 2 weeks of oclacitinib monotherapy; (c) Dog 2: Firm dermal swelling of the right side of the muzzle (arrows) before oclacitinib therapy; (d) Dog 2: Complete lesion resolution after 3 weeks of oclacitinib monotherapy.

**FIGURE 2:**
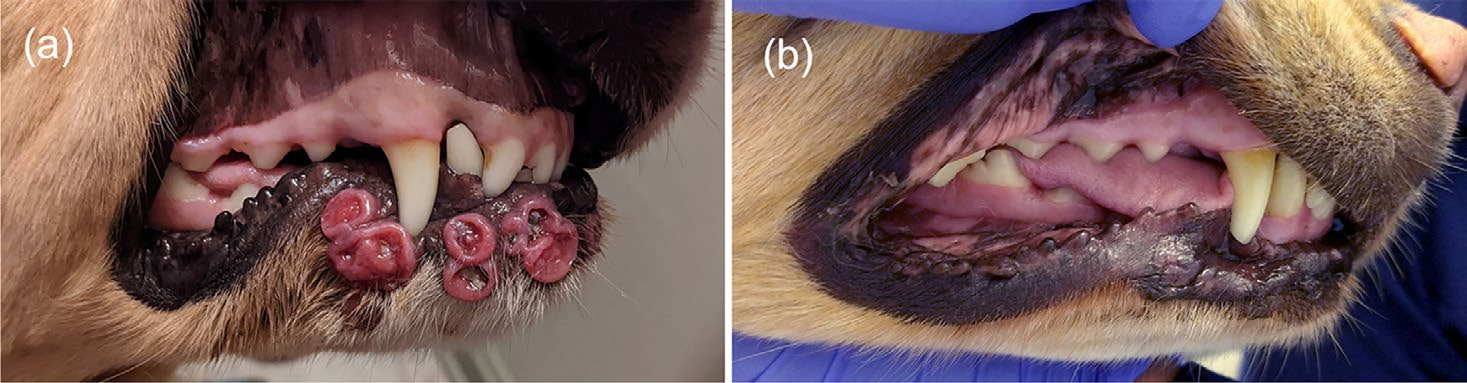
(a) Dog 7: Erythematous, annular plaques with central crateriform ulcers along mandibular lip margins before oclacitinib therapy; (b) Dog 7: Complete lesion remission after 12 weeks of oclacitinib monotherapy.

All dogs presented with multifocal firm dermal nodules and/or erythematous plaques, with variable alopecia, involving the face (periocular skin, muzzle), dorsal head, trunk and/or limbs (Figure 1). An 8‐year‐old spayed female mixed‐breed dog had an acute onset of severe sneezing episodes 2 weeks before onset of firm dermal swelling of the right side of the muzzle (Figure 1), followed by multifocal erythematous nodules on the dorsal head, flanks and lateral thoraces. A 5‐year‐old castrated male mixed‐breed dog initially presented with multifocal firm, annular erythematous plaques, which waxed and waned with tetracycline and niacinamide therapy for > 1 year, followed by development of mild nasal stertor and depigmentation of the nasal planum surrounding the left nare. Eight months after onset of nasal stertor, the cutaneous lesions progressed, together with serous nasal discharge and sneezing. In addition to ulcerated plaques on the distal limbs, a 6‐year‐old spayed female golden retriever presented with erythematous annular plaques with central crateriform ulcers on the lip margins and gingival nodules (Figure 2). A 3‐year‐old castrated male German shorthaired pointer had multifocal firm dermal plaques and nodules over the face, dorsal head, flanks and limbs, in addition to a firm swelling of the oral mucosa surrounding the right maxillary second premolar (tooth 106).

Four of 10 dogs were classified as having cutaneous reactive histiocytosis based on history, physical examination findings, a systemic work‐up (bloodwork, abdominal and thoracic imaging) and/or a complete postmortem examination (performed for one dog). Two dogs did not have a complete diagnostic work‐up performed for investigation of systemic involvement; these dogs had unclassified reactive histiocytosis. The remaining four dogs were classified as having systemic reactive histiocytosis based on history, clinical signs, physical examination findings and/or diagnostic results (advanced imaging, oral biopsies), indicating probable or confirmed involvement of the nasal or oral cavities. None of the dogs had clear evidence of visceral involvement based on clinical signs, physical examination findings or results of a diagnostic work‐up (when available). The diagnostic work‐up and disease classification for each dog is summarised in Table S2.

### 2.3 Histopathological Findings

**FIGURE 3:**
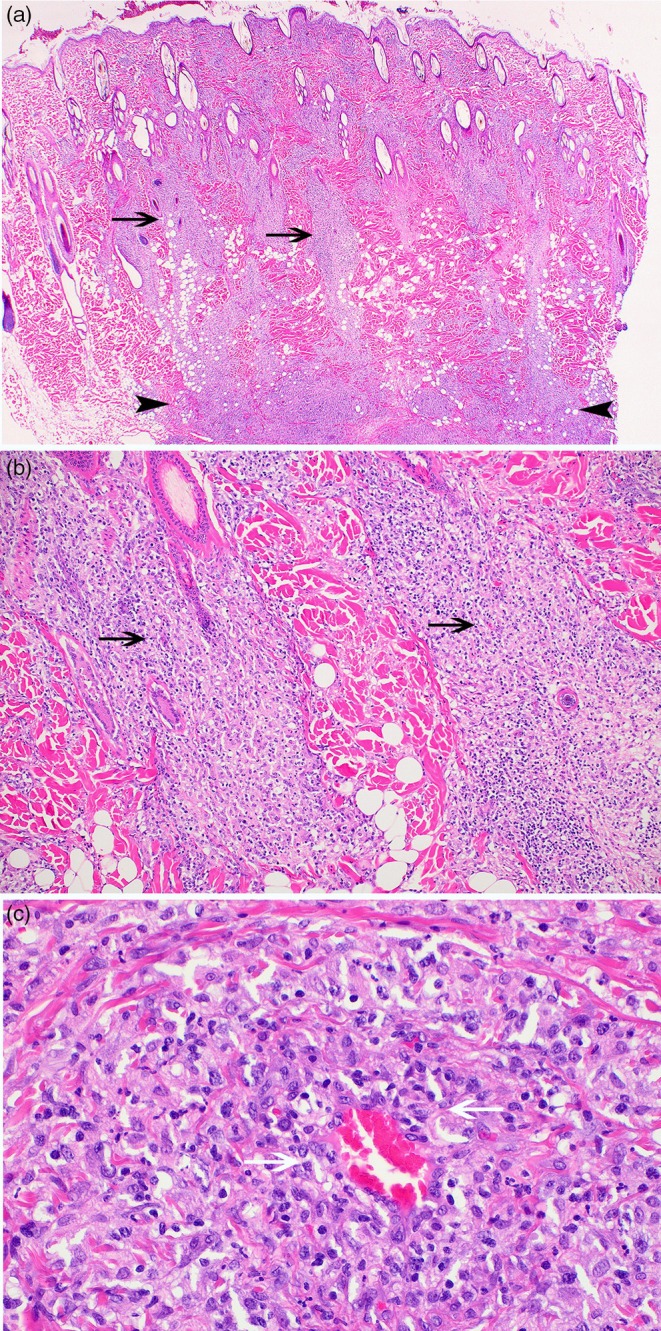
Histological features of skin biopsies from Dog 2 at the time of diagnosis. (a) Nodular‐to‐diffuse infiltrate of histiocytes, small lymphocytes and neutrophils, most prominent within the mid‐ to deep dermis and extending to the superficial subcutis (black arrowheads; ‘bottom‐heavy’ cellular infiltrate). Cellular infiltrate tracks along adnexa to the superficial dermis (black arrows). Haematoxylin & eosin, ×2. (b) Higher magnification view of the nodular infiltrate of histiocytes and small lymphocytes concentrated around and tracking along adnexal structures in the mid‐dermis (black arrows). H&E, ×10. (c) Cellular infiltrate multifocally surrounds deep dermal blood vessels, demonstrating marked angiocentricity (white arrows). H&E, ×40.

**FIGURE 4:**
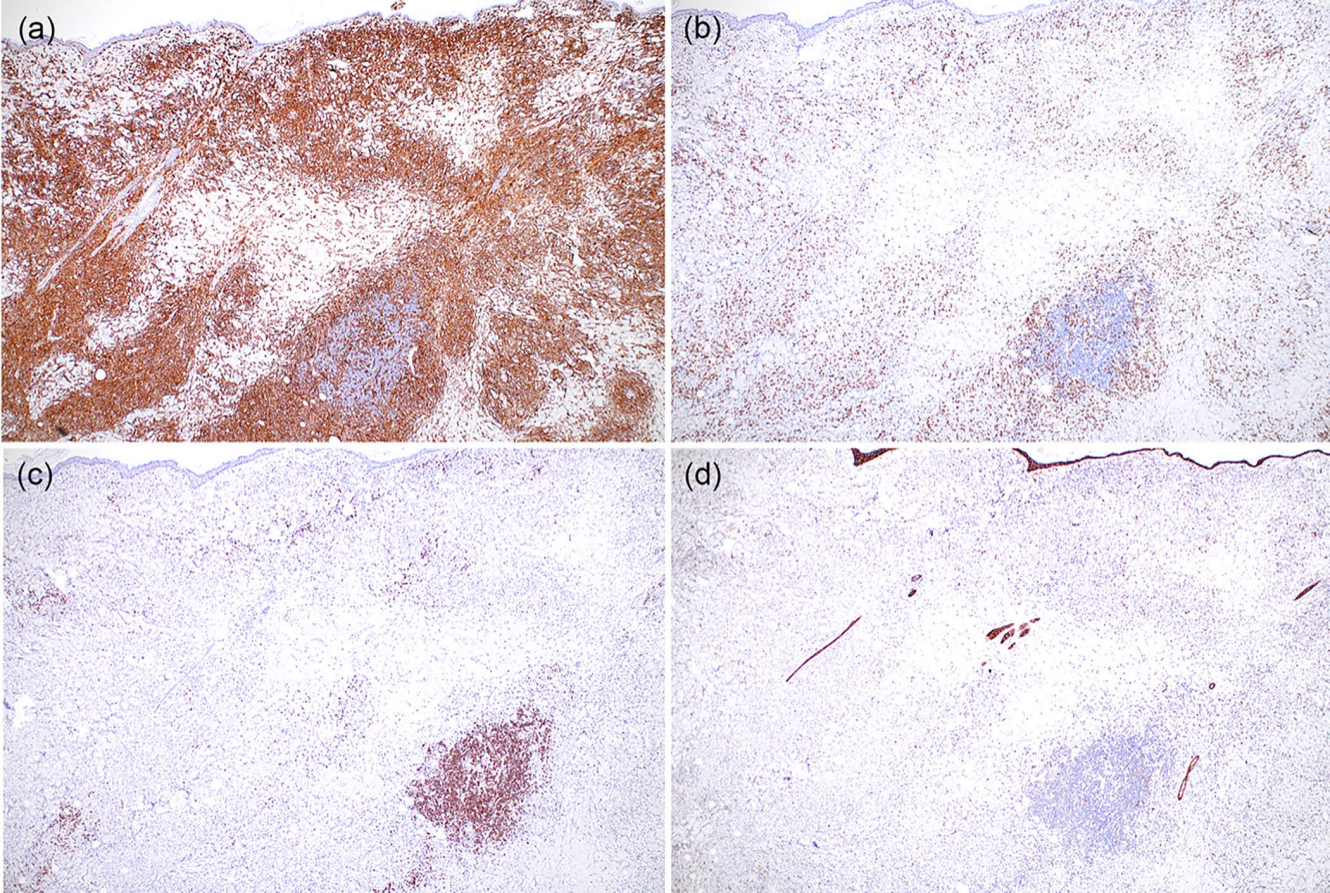
Photomicrographs depicting immunohistochemical features of skin biopsies from Dog 2 at ×4 magnification. (a) Strong diffuse cytoplasmic immunolabelling for Iba‐1 of histiocytes within the dermis. (b) Scattered and nonaggregated T lymphocytes exhibiting positive immunolabelling for CD3 within the dermis. (c) B lymphocytes exhibiting positive immunolabelling for CD79b scattered throughout the dermis and aggregated within a lymphoid follicle. (d) Strong diffuse membranous immunolabelling for E‐cadherin of the epidermis and adnexal epithelium, with scattered positively staining histiocytes within the dermis.

Skin biopsies were collected from multiple plaques or nodules in all dogs, as well as from the firm right‐sided swelling of the dorsal muzzle for the dog depicted in Figure 1. Biopsy collection was performed a median of 2 months (range 5 days–3 years) before initiation of oclacitinib therapy (Table S1). Histological features from skin biopsies of all dogs included a nodular‐to‐diffuse dermal infiltrate of histiocytes, and variable numbers of small lymphocytes and neutrophils, most prominent within the mid‐ to deep dermis and demonstrating marked angiocentricity in some cases (Figure 3). Histiocytes had large round‐to‐ovoid or reniform nuclei and abundant pale eosinophilic cytoplasm. Plasma cells were prominent within the dermal infiltrate in skin biopsies from two dogs (Figure S2). Histochemical stains (Gram, acid fast and Grocott's methenamine silver) were performed in all cases, and no bacteria or fungi were identified. Immunohistochemical stains for lymphocyte markers (CD3 and CD20 or CD79b), ionised calcium binding adapter molecule 1 (Iba‐1) and epithelial cadherin (E‐cadherin) were performed from skin biopsy samples of eight of 10 dogs (Figure 4). In all cases, the dermal infiltrates of histiocytes exhibited strong cytoplasmic staining for Iba‐1. Biopsy samples from six dogs showed scattered and nonaggregated CD3‐positive T cells within the dermis, with more numerous CD3‐positive T cells within the dermal infiltrate for two dogs. In both cases, histiocytes exhibiting marked, diffuse immunolabelling for Iba‐1 predominated within the dermal infiltrate, supporting a diagnosis of reactive histiocytosis rather than inflamed non‐epitheliotropic cutaneous T‐cell lymphoma. Scattered CD79b‐ or CD20‐positive B cells were noted in all cases with occasional aggregates of positively staining cells within lymphoid follicles. Epidermal and adnexal epithelial structures exhibited strong membranous staining for E‐cadherin, while < 15%–20% of dermal histiocytes stained positively in all samples. Molecular clonality analyses of immunoglobulin heavy chain (IgH), immunoglobulin kappa light chain (IgK) and T‐cell receptor gamma (TRG) loci were performed from skin biopsy samples of one dog (2‐year‐old castrated male Doberman pinscher). These analyses demonstrated polyclonal arrangements of B‐cell and T‐cell loci, consistent with a reactive/inflammatory process.

Three dogs also had biopsies collected from the oral cavity. One of these dogs, an 8‐year‐old spayed female mixed‐breed dog, had an incisional biopsy collected from the oral mucosa surrounding the right maxillary canine tooth (tooth 104); soft tissue swelling of this region was noted on a computed tomography (CT) scan of the head. The second dog, a 6‐year‐old spayed female Golden retriever, had biopsies collected from the upper and lower lips, as well as the gingiva, and the third dog, a 3‐year‐old castrated male German shorthaired pointer, had an incisional biopsy collected from the oral mucosa surrounding tooth 106. Lymphohistiocytic proliferation also was noted from the oral mucosa, lips and/or gingiva in all three dogs. As seen in the haired skin, the cellular infiltrate was ‘bottom‐heavy’ and contained predominantly histiocytes with intermixed lymphocytes without granuloma formation. Nasal biopsies were not performed for the two dogs who exhibited nasal stertor and sneezing.

### 2.4 Clinicopathological Findings

A complete blood count (CBC) and biochemical profile were performed for eight of 10 dogs at the onset of skin lesions. Results were unremarkable for nine dogs, while an 8‐year‐old castrated male Pembroke Welsh corgi's biochemical profile initially showed mild hypoalbuminaemia (2.1 g/dL; normal 2.7–4.4 g/dL) and hyperglobulinaemia (6.1 g/dL; normal 1.6–3.6 g/dL). A biochemical profile was repeated after 8 weeks of oclacitinib therapy and full resolution of skin lesions; these abnormalities also were resolved at that time.

### 2.5 Imaging

Thoracic radiographs and abdominal ultrasound were performed for four of 10 dogs as part of a systemic work‐up at the onset of skin lesions; they were unremarkable for all dogs. Two dogs underwent CT scans of the head and nasal cavity. For one of these dogs (8‐year‐old spayed female mixed‐breed dog), the CT scan was performed as part of the work‐up for acute sneezing episodes and before the onset of firm right‐sided swelling of the dorsal muzzle. This dog's CT scan showed focal soft tissue swelling of the left nare and nasal septum, mild mucosal thickening and fluid accumulation within the nasal passages, and subcutaneous soft tissue swelling adjacent to the root of the right maxillary canine tooth (tooth 104). For the other dog (5‐year‐old castrated male mixed‐breed dog), the CT scan was performed following progression of skin lesions (erythematous annular plaques), nasal stertor and sneezing. Skin biopsies were collected contemporaneously. This dog's CT scan showed soft tissue swelling and mucosal thickening of the rostral nasal cavity.

### 2.6 Treatment

Full treatment information for each dog is summarised in Table S2. Treatments employed before treatment with oclacitinib included doxycycline, tetracycline, or minocycline with niacinamide (six of 10 dogs), prednisone (five of 10 dogs), ciclosporin with or without ketoconazole (three of 10 dogs), mycophenolate mofetil (two of 10 dogs), azathioprine (two of 10 dogs), and tacrolimus ointment (one dog). Three dogs did not receive treatment for reactive histiocytosis before initiation of oclacitinib therapy.

### 2.7 Outcome of Oclacitinib Therapy

Full information regarding the outcome of oclacitinib therapy for each dog is summarised in Table S2. Seven of 10 dogs initially received oclacitinib twice daily at a median dosage of 0.46 mg/kg (range 0.4–0.69 mg/kg). For the remaining three dogs, oclacitinib therapy was initiated once daily (at 0.5, 0.59 and 0.69 mg/kg, respectively). Each dog achieved full resolution of skin lesions while receiving oclacitinib (noted in medical records between 2 and 12 weeks following initiation of oclacitinib therapy), and lesion remission was maintained with oclacitinib monotherapy with varying follow‐up times. Nine of 10 dogs received oclacitinib for maintenance of lesion remission once daily at a median dosage of 0.46 mg/kg (range 0.31–0.61 mg/kg). The remaining dog received oclacitinib at a dosage of 0.39 mg/kg twice daily for maintenance of lesion remission. Four dogs experienced brief recurrences of dermal plaques and nodules (accompanied by sneezing for one dog), which were responsive to temporary increases in oclacitinib dosage. The recurrence of skin lesions for one of these dogs, a 6‐year‐old spayed female Golden retriever, coincided with a reduction of oclacitinib dosing frequency to every‐other‐day.

Four of 10 dogs were followed to the point of humane euthanasia; the remaining six dogs were alive at the time of writing this report (Table S2). The median age at the time of euthanasia for these four dogs was 8.75 years (range 3–10 years) and the median duration of disease at the time of euthanasia was 2.3 years (range 1–5.5 years). One of these dogs, a 2‐year‐old castrated male Doberman pinscher (Figure S1), was treated with oclacitinib for only 3 weeks before humane euthanasia as a consequence of acute small intestinal obstruction following ingestion of a foreign body (this dog's third obstructive foreign body). A complete necropsy was performed; grossly, multifocal alopecia with no dermal plaques was noted. Histopathological findings of the skin showed dermal fibrosis with little evidence of the previous nodular lymphohistiocytic and plasmacytic dermatitis (Figure S2). The second dog, a 7‐year‐old spayed female Bernese mountain dog, was euthanised 2.5 years after initiation of oclacitinib therapy as a consequence of progression of a mediastinal mast cell tumour. A third dog, a 10‐year‐old castrated male Pembroke Welsh corgi, was euthanised 2 years after initiation of oclacitinib therapy as a consequence of haemoabdomen secondary to a ruptured splenic mass. Two years after initiation of oclacitinib therapy, a 10‐year‐old spayed female mixed‐breed dog was euthanised as a consequence of rapidly progressive inappetence, lethargy, vomiting, muscle tremors and trigeminal neuropathy. A few dermal nodules on the dog's head and pinnae also were noted concomitant with the onset of these clinical signs. A postmortem examination was not performed and it is unknown whether this dog's clinical deterioration was related to systemic progression of reactive histiocytosis or another disease process.

## 3 Discussion

Dogs in this case series showed a positive response to treatment with oclacitinib for both induction and maintenance of lesion remission. Reactive histiocytosis is reported to have a naturally waxing and waning course, with the potential for spontaneous remission, making it difficult to fully assess therapeutic response [1, 2]. One dog, for example, was diagnosed with systemic reactive histiocytosis based on skin and oral mucosal biopsies; yet therapy was not instituted for 1.5 years, when progression of skin lesions prompted evaluation by a referral clinician. Most dogs in this case series (seven of 10), however, experienced lack of disease control or progression while receiving other immunomodulatory therapies. Notably, three of these dogs were previously treated with four or more immunomodulatory agents following histological confirmation of reactive histiocytosis, suggesting that durable remission without therapy would not be expected and supporting a true positive response to oclacitinib therapy. A previous study of 32 dogs with cutaneous reactive histiocytosis reported potential disease recurrences with attempts to taper or withdraw immunomodulatory medications and emphasised the importance of maintenance therapy for long‐term disease control [3]. Likewise, in this case series, one dog experienced recurrence of dermal nodules with the attempt to reduce oclacitinib to an every‐other‐day dosing frequency. While a limitation of this retrospective study was the lack of consistent timing of reexaminations to assess treatment response, oclacitinib appears to have rapid efficacy for induction of marked lesion improvement or remission in reactive histiocytosis, as has been reported for other immune‐mediated dermatoses [6, 11, 12, 14]. Six of 10 dogs had complete lesion remission or marked lesion improvement noted within 4 weeks of instituting oclacitinib therapy. Furthermore, gross lesion remission was substantiated by the lack of histological evidence of reactive histiocytosis in skin samples collected at the time of necropsy for one dog (3 weeks after instituting oclacitinib therapy).

The immunological mechanisms of canine reactive histiocytosis have not been fully established, yet pro‐inflammatory cytokines and T‐cell‐derived cytokines involved in the proliferation and activation of dendritic cells, including tumour necrosis factor alpha (TNFα), IL‐6, IL‐12 and interferon gamma (IFNɣ), may contribute to lesion development and/or maintenance [3]. Lymphocytic infiltrates within lesional skin are rich in CD8+ T lymphocytes [1, 2]. The efficacy of oclacitinib for clinical management of reactive histiocytosis may be a consequence of its effects on cytokine signalling and T‐cell populations. Inhibition of interferon signalling pathways by oclacitinib may particularly contribute to its efficacy for management of hyperkeratotic erythema multiforme [6], ischaemic dermatopathy [7] and variants of canine chronic cutaneous lupus erythematosus [12], as well as reactive histiocytosis. Results of in vitro and in vivo analyses of the immunomodulatory effects of oclacitinib have varied. A previous study demonstrated that oclacitinib inhibited T‐helper 2 associated pro‐inflammatory and pro‐pruritic cytokines (IL‐2, IL‐6, IL‐4, IL‐13 and IL‐31) in vitro [15]. Another in vitro study, however, showed that oclacitinib did not inhibit cytokines associated with proliferation and activation of T cells (IL‐2 and IL‐15) or T‐helper 1 associated pro‐inflammatory cytokines (IL‐18, IFNɣ, TNFα) at concentrations typically achieved in the plasma with standard oral dosing (0.4–0.6 mg/kg) [16]. Likewise, no in vivo effect on cytokine production from antigen‐stimulated T cells of atopic dogs treated with oclacitinib at the label dosage for 12 months was demonstrated [17]. Shifts in T‐cell populations may occur during oclacitinib therapy. An in vitro study showed that oclacitinib promoted apoptosis and depletion of canine CD4+ and CD8+ T cells [18], yet these results have not been replicated in vivo in mice or dogs [17, 19]. Studies in atopic dogs treated with oclacitinib at the label dosage have shown significant increases in CD4+ T cells over time [17, 20], including Foxp3 + CD4+ regulatory T cells, although the long‐term immunomodulatory effects in non‐atopic dogs are unknown [20]. Higher plasma concentrations of oclacitinib, equivalent to oral doses of 3–4 mg/kg twice daily, may be associated with increased immunosuppression. At these concentrations, significant reductions in T‐cell proliferation and production of IL‐2, IL‐15, IFNɣ and IL‐18 were noted [16]. For the most part, the dogs in this case series received oclacitinib within or just above the high end of the label dose range. While one dog received a higher dosage (0.93 mg/kg once daily) for 4 weeks, this is still below this putative immunosuppressive dosage (3–4 mg/kg twice daily) [16]. Taken together, further work is needed to elucidate the immunological mechanisms of oclacitinib for management of reactive histiocytosis and other immune‐mediated dermatoses.

Four of 10 dogs in this case series were diagnosed with cutaneous reactive histiocytosis, and four dogs were diagnosed with probable systemic reactive histiocytosis based on suspected nasal mucosal (two dogs), or histologically confirmed oral mucosal and/or gingival involvement. Although the two dogs did not have nasal biopsies collected, involvement of the nasal cavity was strongly suspected owing to clinical signs (sneezing, nasal stertor) and CT findings (nasal mucosal thickening). In a prior study, involvement of the nasal cavity was fairly common in dogs with systemic reactive histiocytosis (occurring in 14 of 26 dogs), while involvement of the oral cavity was uncommon (gingival lesions were noted in just one dog) [2]. To the best of the authors' knowledge, reports of oral mucosal involvement in canine reactive histiocytosis are rare, with a single published report of sublingual disease [21]. The results of the present case series suggest that oral mucosal lesions may not be as uncommon as presumed (occurring in three of 10 dogs in this report) and that a careful oral examination should be performed in all dogs with a known or suspected diagnosis of reactive histiocytosis.

Systemic reactive histiocytosis has been reported to be less responsive to corticosteroid therapy, to have a more aggressive disease course, and to be more likely to result in humane euthanasia than cutaneous reactive histiocytosis [1, 2]. In a previous study of 32 dogs with cutaneous reactive histiocytosis, dogs with nasal planum involvement were found to experience more recurrences [3]. This previous study suggested that nasal planum involvement may represent a more aggressive or refractory form of the disease [3], yet it also is possible that dogs with nasal planum lesions may be more likely to have occult nasal mucosal involvement. In the present case series, the dogs with suspected or known nasal or oral mucosal involvement were not noted to be less responsive to oclacitinib or to have notably different outcomes with the exception of one dog (Dog 2; an 8‐year‐old spayed female mixed‐breed dog). This dog's reactive histiocytosis was well‐managed with oclacitinib for 2 years before rapidly progressive clinical signs of systemic illness were noted together with a few new cutaneous nodules. Systemic progression of reactive histiocytosis was suspected yet was not confirmed by postmortem examination. Results of this case series suggest that mucosal involvement of reactive histiocytosis is not necessarily a poor prognostic indicator and may not progress to more widespread involvement of internal organs, although it is possible that this dog had a more biologically aggressive form of the disease. In a previous report of sublingual reactive histiocytosis, the dog's lesions were successfully managed for 10 months with tetracycline and niacinamide with no recurrence after therapeutic withdrawal for a 2 year follow‐up period [21]. The authors of this prior report suggested that localised mucosal reactive histiocytosis may represent a variant of cutaneous disease rather than a manifestation of systemic reactive histiocytosis [21].

In conclusion, oclacitinib was an effective therapy for both induction of lesion remission and maintenance of canine reactive histiocytosis with both skin and mucosal involvement in this case series. Further studies, as well as randomised clinical trials, are needed to clarify the immunological mechanisms and to optimise dosing protocols of JAK inhibitors for management of immune‐mediated dermatoses in veterinary patients.

## Author Contributions

C.L.C. and E.A.M. conceptualised the study, with input and assistance from A.L. C.L.C. reviewed medical records and collected data with assistance from E.A.M. and A.L. E.A.M. reviewed all skin biopsies and confirmed histological diagnoses. C.L.C. wrote the manuscript with input, assistance and approval from E.A.M. and A.L.

## Funding

This study was self‐funded.

## Conflicts of Interest

The authors declare no conflicts of interest.

## Supporting information


**Figure S1:** Clinical images of Dog 3 before oclacitinib therapy depicting (a) nonlesional face and head and (b) multifocal coalescing alopecic plaques over mid‐ to caudal dorsum with more discrete alopecic dermal nodules and plaques over lateral aspect of left hind limb. Adhesive bandages cover skin biopsy sites.

**Figure S2:** Histological features of skin biopsies from Dog 3 at the time of diagnosis (a) and postmortem (b) following humane euthanasia for acute small intestinal obstruction, 3 weeks after starting oclacitinib monotherapy. Biopsies were collected initially from coalescing dermal plaques and postmortem from areas of alopecia (dermal plaques had resolved). (a) Dense infiltrate of histiocytes, plasma cells, and lymphocytes extends throughout the dermis to the subcutis, and is particularly prominent within the mid‐ to deep dermis (‘bottom‐heavy’ cellular infiltrate). Haematoxylin & eosin, ×4. Inset: dermal cellular infiltrate at higher magnification (×10). (b) Dermal fibrosis is noted, with marked reduction of the previous nodular‐to‐diffuse dermal‐to‐subcutaneous infiltrates of histiocytes, plasma cells and lymphocytes. H&E, ×4.

**Table S1:** Signalment information, duration of clinical signs at initiation of oclacitinib therapy and timing of skin biopsy collection before initiation of oclacitinib therapy for 10 dogs with reactive histiocytosis.

**Table S2:** Treatment information, diagnostics performed, classification of reactive histiocytosis (cutaneous, systemic or unclassified), follow‐up time after initiation of oclacitinib therapy and outcome for 10 dogs.

## Data Availability

The data that support the findings of this study are available from the corresponding author upon reasonable request.
