# Supplementary figures and images for: Treatment of Reactive Histiocytosis With Oclacitinib: A Retrospective Case Series of 10 Dogs
Source: Vet Dermatol. 2026 Jan 28;37(3):419–26. doi: 10.1111/vde.70048 (PMC13167641; doi:10.1111/vde.70048)

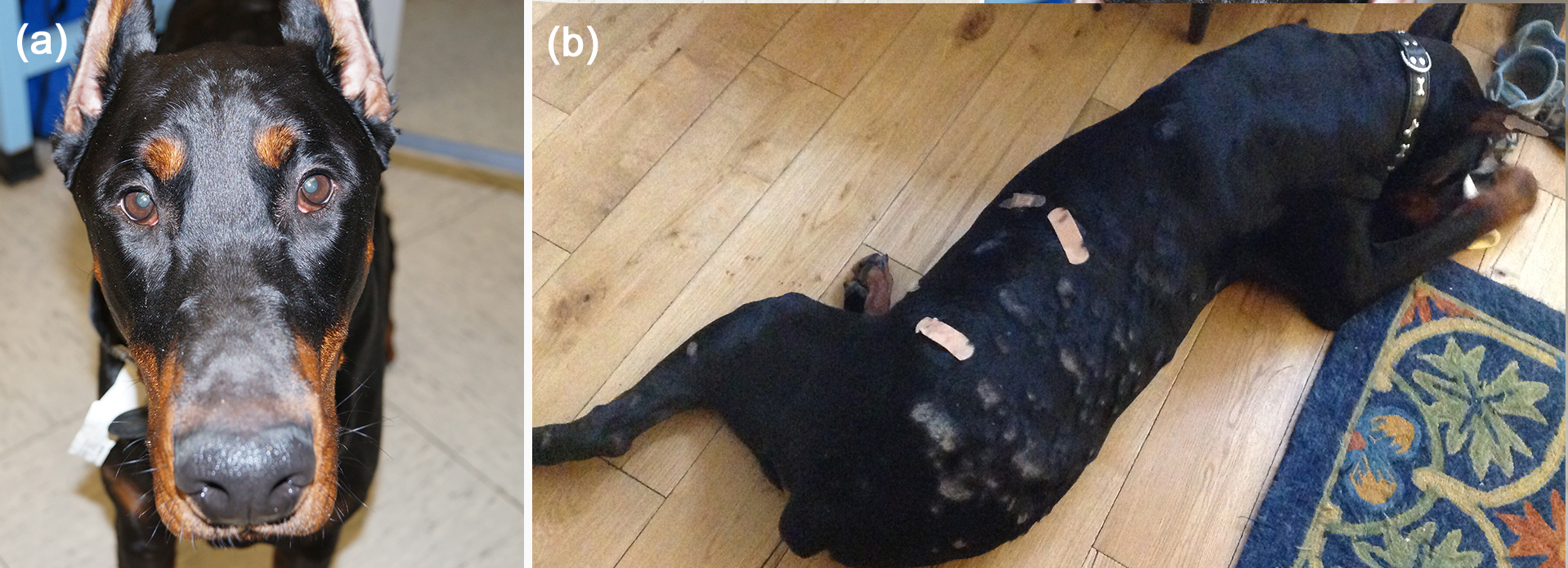

Supplement: Supplementary file 1 — Figure S1: Clinical images of Dog 3 before oclacitinib therapy depicting (a) nonlesional face and head and (b) multifocal coalescing alopecic plaques over mid‐ to caudal dorsum with more discrete alopecic dermal nodules and plaques over lateral aspect of left hind limb. Adhesive bandages cover skin biopsy sites. [file VDE-37-419-s001.jpg]

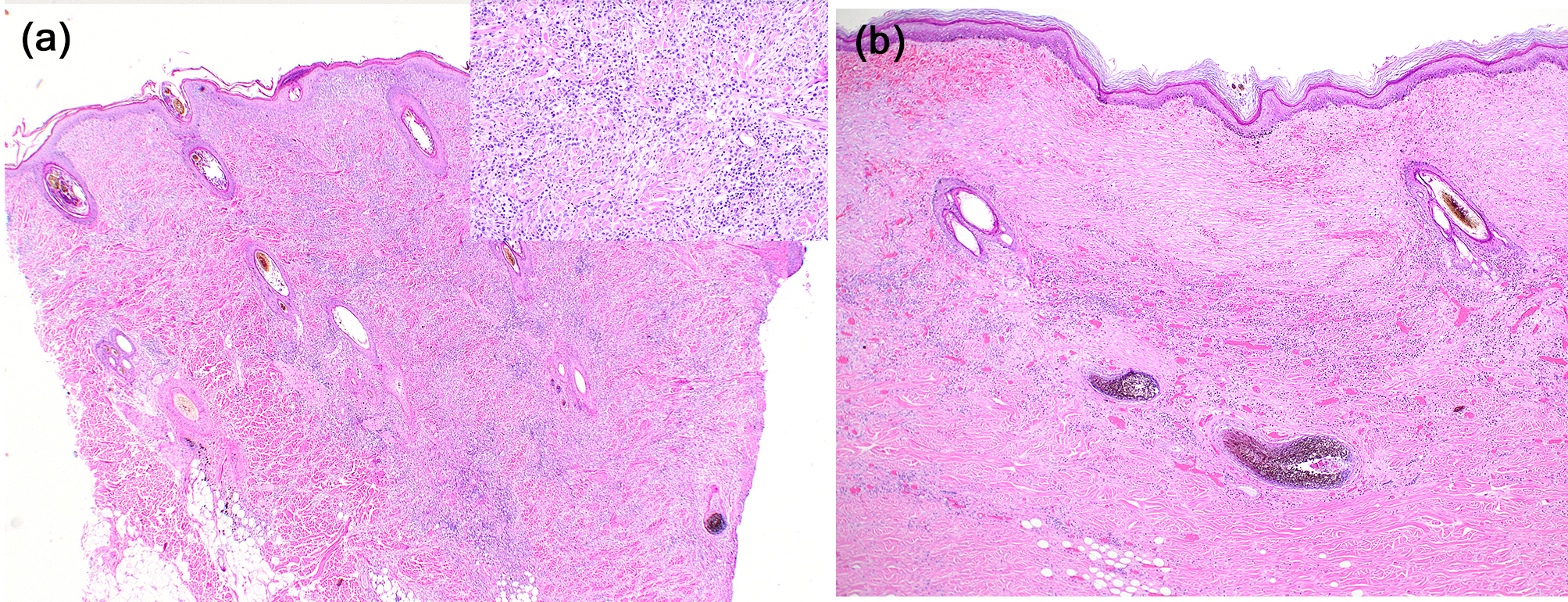

Supplement: Supplementary file 2 — Figure S2: Histological features of skin biopsies from Dog 3 at the time of diagnosis (a) and postmortem (b) following humane euthanasia for acute small intestinal obstruction, 3 weeks after starting oclacitinib monotherapy. Biopsies were collected initially from coalescing dermal plaques and postmortem from areas of alopecia (dermal plaques had resolved). (a) Dense infiltrate of histiocytes, plasma cells, and lymphocytes extends throughout the dermis to the subcutis, and is particularly prominent within the mid‐ to deep dermis (‘bottom‐heavy’ cellular infiltrate). Haematoxylin & eosin, ×4. Inset: dermal cellular infiltrate at higher magnification (×10). (b) Dermal fibrosis is noted, with marked reduction of the previous nodular‐to‐diffuse dermal‐to‐subcutaneous infiltrates of histiocytes, plasma cells and lymphocytes. H&E, ×4. [file VDE-37-419-s002.jpg]
