# Supplementary material for: Treatment of Reactive Histiocytosis With Oclacitinib: A Retrospective Case Series of 10 Dogs
Source: Vet Dermatol. 2026 Jan 28;37(3):419–26. doi: 10.1111/vde.70048 (PMC13167641; doi:10.1111/vde.70048)
Supplement: Supplementary file 3 — Table S1: Signalment information, duration of clinical signs at initiation of oclacitinib therapy and timing of skin biopsy collection before initiation of oclacitinib therapy for 10 dogs with reactive histiocytosis. [file VDE-37-419-s003.docx]

**Table S1**

| **Dog** | **Breed** | **Age (years)** | **Sex** | **Weight** | **Duration of clinical signs at initiation of oclacitinib therapy** | **Timing of skin biopsy collection before initiation of oclacitinib therapy** |
| --- | --- | --- | --- | --- | --- | --- |
| 1 | Bernese mountain dog | 5 | FS | 39.3 | 3 years | 3 years |
| 2 | Mixed breed | 8 | FS | 17.3 | 3 months | 2 months |
| 3 | Doberman pinscher | 2 | MC | 52.9 | 1 year | 1 year |
| 4 | Pembroke Welsh corgi | 8 | MC | 12.3 | 4 months | 19 days |
| 5 | Mixed breed | 5 | MC | 46.1 | 2 years | 14 days |
| 6 | Shetland sheepdog | 3 | FS | 13.5 | 1 year | 5 days |
| 7 | Golden retriever | 6 | FS | 23.0 | 7 months | 5 months |
| 8 | Labrador retriever | 3 | FS | 40.0 | 5 months | 2 months |
| 9 | Golden retriever | 2 | MC | 40.0 | 6 months | 6 months |
| 10 | German shorthaired pointer | 3 | MC | 31.1 | 1.5 years | 1.5 years |

FS, female spayed; MC, male castrated
