# Supplementary material for: Treatment of Reactive Histiocytosis With Oclacitinib: A Retrospective Case Series of 10 Dogs
Source: Vet Dermatol. 2026 Jan 28;37(3):419–26. doi: 10.1111/vde.70048 (PMC13167641; doi:10.1111/vde.70048)
Supplement: Supplementary file 4 — Table S2: Treatment information, diagnostics performed, classification of reactive histiocytosis (cutaneous, systemic or unclassified), follow‐up time after initiation of oclacitinib therapy and outcome for 10 dogs. [file VDE-37-419-s004.docx]

**Table S2**

| **Dog** | **Cutaneous, systemic, or unclassified disease** | **Diagnostics performed** | **Prior therapies** | **Oclacitinib dose (induction)** | **Oclacitinib dose (maintenance)** | **Outcome of oclacitinib therapy** | **Follow-up time; patient outcome** |
| --- | --- | --- | --- | --- | --- | --- | --- |
| 1 | Cutaneous | CBC, biochemical profile, thoracic radiographs, abdominal ultrasound, IHC | Doxycycline & niacinamide; ciclosporin & ketoconazole; prednisone; mycophenolate mofetil; azathioprine | 0.61 mg/kg twice daily for 2 weeks | 0.61 mg/kg once daily | Full resolution of skin lesions within 8 weeks, continued remission with oclacitinib alone | 2.5 years; humane euthanasia as a consequence of progression of mediastinal mast cell tumour |
| 2 | Systemic (oral mucosal/suspected nasal mucosal involvement) | CBC, biochemical profile, head/nasal CT, oral mucosal biopsy, IHC | Doxycycline & niacinamide; prednisone | 0.46 mg/kg twice daily for 2 weeks | 0.46 mg/kg once daily | Full resolution of skin lesions and sneezing within 3 weeks. Two brief flares in sneezing, firm right-sided muzzle swelling, and dermal nodules managed with increase in oclacitinib dosing frequency to twice daily for 2 weeks | 2 years; humane euthanasia as a consequence of progressive lethargy, inappetence, muscle tremors, vomiting, trigeminal neuropathy (necropsy not performed) |
| 3 | Cutaneous | CBC, biochemistry profile, complete necropsy, IHC, Molecular clonality analyses | Ciclosporin & ketoconazole; prednisone; azathioprine; mycophenolate mofetil | 0.45 mg/kg twice daily for 2 weeks | 0.45 mg/kg once daily | Full resolution of skin lesions within 3 weeks with oclacitinib alone; humane euthanasia following intestinal obstruction | 3 weeks; humane euthanasia as a consequence of small intestinal obstruction (complete necropsy performed) |
| 4 | Cutaneous | CBC, biochemistry profile, thoracic radiographs, abdominal ultrasound, IHC | NA | 0.44 mg/kg twice daily for 2 weeks | 0.44 mg/kg once daily | Full resolution of skin lesions within 2 weeks, continued remission with oclacitinib alone | 2 years; humane euthanasia as a consequence of haemoabdomen secondary to ruptured splenic mass (necropsy not performed) |
| 5 | Systemic (suspected nasal mucosal involvement) | Head/nasal CT, IHC | Tetracycline & niacinamide, prednisone | 0.69 mg/kg once daily for 9 months | 0.52 mg/kg once daily | Full resolution of skin lesions, sneezing and nasal stertor within 4 weeks, continued remission with oclacitinib alone and continued remission following oclacitinib dose reduction | 1 year; alive and in disease remission at time of report |
| 6 | Unclassified | IHC | NA | 0.59 mg/kg once daily | 0.59 mg/kg once daily | Full resolution of skin lesions within 4 weeks, continued remission with oclacitinib alone | 5 months; alive and in disease remission at time of report |
| 7 | Systemic (oral mucosal and gingival involvement) | CBC, biochemistry profile, oral mucosal/gingival biopsies, IHC | Minocycline & niacinamide; prednisone; ciclosporin; tacrolimus ointment | 0.69 mg/kg twice daily (duration of induction dose not recorded) | 0.31 mg/kg once daily | Marked lesion improvement within 2 weeks, full resolution of skin and oral mucosal lesions within 3 months; oclacitinib gradually tapered over 6 months with recurrence of dermal nodules at 0.31 mg/kg every 48 h. Oclacitinib dose increased to 0.93 mg/kg once daily for 4 weeks, then 0.62 mg/kg once daily for 8 weeks, then to 0.31 mg/kg once daily thereafter; continued remission with oclacitinib alone | 1 year; alive and in disease remission at time of report |
| 8 | Cutaneous | CBC, biochemistry profile, thoracic radiographs, abdominal ultrasound, IHC | Doxycycline & niacinamide | 0.4 mg/kg twice daily for 10 months | 0.4 mg/kg once daily | Full resolution of skin lesions within 6 weeks, continued remission with oclacitinib alone | 1.5 years; alive and in disease remission at time of report |
| 9 | Unclassified | CBC, biochemistry profile | Doxycycline & niacinamide | 0.5 mg/kg once daily | 0.57 mg/kg once daily | Full resolution of skin lesions within 6 weeks; recurrence of dermal nodules 2 months later, managed with increase in oclacitinib dosing frequency to twice daily for 2 weeks | 5 months; alive and in disease remission at time of report |
| 10 | Systemic (oral mucosal involvement) | CBC, biochemistry profile, oral mucosal biopsy | NA | 0.52 mg/kg twice daily for 4 months | 0.39 mg/kg twice daily | Full resolution of skin lesions within 6 weeks; recurrence of dermal plaques noted when oclacitinib dose reduced to 0.52 mg/kg once daily after 4 months. Oclacitinib dose increased back to 0.52 mg/kg twice daily for 6 months, then reduced to 0.39 mg/kg twice daily; continued remission with oclacitinib alone | 1 year; alive and in disease remission at time of report |

NA, not applicable; CBC, complete blood count; CT, computed tomography; IHC, immunohistochemical investigation for Iba-1, CD3, CD79b or CD20, E-cadherin
